# Computational Analysis of Viscoelastic Properties of Crosslinked Actin Networks

**DOI:** 10.1371/journal.pcbi.1000439

**Published:** 2009-07-17

**Authors:** Taeyoon Kim, Wonmuk Hwang, Hyungsuk Lee, Roger D. Kamm

**Affiliations:** 1 Department of Mechanical Engineering, Massachusetts Institute of Technology, Cambridge, Massachusetts, United States of America; 2 Department of Biomedical Engineering, Texas A&M University, College Station, Texas, United States of America; 3 Department of Biological Engineering, Massachusetts Institute of Technology, Cambridge, Massachusetts, United States of America; University of California, Berkeley, United States of America

## Abstract

Mechanical force plays an important role in the physiology of eukaryotic cells whose dominant structural constituent is the actin cytoskeleton composed mainly of actin and actin crosslinking proteins (ACPs). Thus, knowledge of rheological properties of actin networks is crucial for understanding the mechanics and processes of cells. We used Brownian dynamics simulations to study the viscoelasticity of crosslinked actin networks. Two methods were employed, bulk rheology and segment-tracking rheology, where the former measures the stress in response to an applied shear strain, and the latter analyzes thermal fluctuations of individual actin segments of the network. It was demonstrated that the storage shear modulus (*G*′) increases more by the addition of ACPs that form orthogonal crosslinks than by those that form parallel bundles. In networks with orthogonal crosslinks, as crosslink density increases, the power law exponent of *G*′ as a function of the oscillation frequency decreases from 0.75, which reflects the transverse thermal motion of actin filaments, to near zero at low frequency. Under increasing prestrain, the network becomes more elastic, and three regimes of behavior are observed, each dominated by different mechanisms: bending of actin filaments, bending of ACPs, and at the highest prestrain tested (55%), stretching of actin filaments and ACPs. In the last case, only a small portion of actin filaments connected via highly stressed ACPs support the strain. We thus introduce the concept of a ‘supportive framework,’ as a subset of the full network, which is responsible for high elasticity. Notably, entropic effects due to thermal fluctuations appear to be important only at relatively low prestrains and when the average crosslinking distance is comparable to or greater than the persistence length of the filament. Taken together, our results suggest that viscoelasticity of the actin network is attributable to different mechanisms depending on the amount of prestrain.

## Author Summary

The actin cytoskeleton provides structural integrity to a cell, is highly dynamic, and plays a central role in a wide variety of phenomena such as migration and the sensation of external forces. For years, researchers have studied the mechanics of the cytoskeleton by creating actin gels in the laboratory in combination with proteins that bridge between and reinforce the actin gel found inside cells. These gels, however, failed to replicate many aspects of cell behavior. Recent studies have shown that tension within the cytoskeleton contributes to the observed stiffness of cells. Still, our understanding of cytoskeletal mechanics is incomplete, and many observed phenomena cannot be explained by existing models. Here, we simulate a three-dimensional network containing actin filaments linked together by other proteins. We studied the relative contributions of thermal fluctuations of the network and the stiffness of filaments and linking proteins. Under conditions that replicate those in a cell, properties of the linking proteins are surprisingly significant, as is the stiffness of the actin filament to stretching. Thermal fluctuations are relatively unimportant, but become more so at low levels of resting tension. At high tensions, a small fraction of filaments support a majority of the load.

## Introduction

Actin is the most abundant intracellular protein in eukaryotic cells and plays an important role in a wide range of biological and mechanical phenomena [1]. Monomeric actin (G-actin) self-assembles to a filamentous form, F-actin, which is crosslinked into the actin cytoskeleton by various actin crosslinking proteins (ACPs). It has been known that mechanical force plays a crucial role in the physiology of eukaryotic cells [2], and therefore appropriate functions of living cells are attributable to the rigorous control of their rheological properties [3]. Thus, investigating rheological properties of actin networks is indispensable for elucidating the mechanics of cells as well as for understanding a wide variety of cellular processes. Experiments have been conducted to probe viscoelastic properties of cells and reconstituted actin gels using a variety of techniques such as microbead rheology, magnetic bead cytometry, and bulk rheology [4]–[23]. In experiments, discrepancies have been observed among measurements using dissimilar methodologies, and many of the observed features are not well understood. For example, viscoelastic moduli measured by single-bead passive microbead rheology are much smaller than those determined by 2-point microrheology or bulk rheology [4]–[6]. Also, although distinct power law responses of the storage modulus have often been observed *in vivo* and *in vitro* [7]–[11], their origin is not yet clearly understood.

Concurrently, characteristics of semi-flexible polymer networks have been studied theoretically and computationally [24]–[34]. Two- [24],[25] and 3-dimensional computational models [26] studying affine and nonaffine deformations of semi-flexible networks responding to large shear strain revealed two regimes dominated by bending or stretching of filaments, respectively. Recently, using a microstructure-based continuum mechanics approach, Palmer and Boyce reproduced many of the rheological properties of actin networks observed in experiments [27]. The viscoelastic behavior of semi-flexible networks was also investigated using dissipative particle dynamics and the concept of microbead rheology, where scale-free behavior of the bead displacement was observed [28],[29]. To date, however, most of these models neither explicitly take into account ACP mechanics nor systematically account for thermal fluctuations, nor have they been used to explore the effects of finite prestress on viscoelasticity, all of which are potentially important factors governing matrix viscoelasticity.

With the objective of extending these previous works and providing new insights into underlying mechanisms, we develop a Brownian dynamics (BD) model of the actin network that includes features such as steric interaction among filaments, the usage of explicit crosslinkers, a more realistic morphology, and the consideration of crosslinker stiffness. By measuring stress in response to applied oscillatory shear strain (“bulk rheology”) and thermal fluctuations of individual segments in the polymeric chain (“segment-tracking rheology”), we investigate viscoelastic properties of actin-like networks. Throughout this paper, for convenience, the term, “actin network” is used to refer to the network being simulated. It should be noted, however, that some of the properties employed in our model, especially for ACPs, were estimated since they are not well-known experimentally. Due to simplifications in the model and parameter uncertainty, the results should therefore be viewed as representative of a generic crosslinked network, but lack a quantitatively precise correspondence to actin networks.

Nevertheless, we found features that semi-quantitatively capture experimentally observed behaviors of actin networks. The storage and loss moduli, *G*′ and *G*″, followed power laws as functions of the oscillation frequency. As the prestrain increased, the network became increasingly elastic. Bending and extensional stiffnesses of actin filaments and ACPs played an important role depending on the degree of prestrain. We found that the mechanical response of the network is dominated by a percolating ‘supportive framework,’ while other actin filaments contribute little to the viscoelastic moduli. Surprisingly, in typical physiological conditions where the distance between crosslinking points along F-actin is much shorter than the actin persistence length, we found that thermal fluctuation plays little role in viscoelasticity, so that the network consisting of crosslinked F-actins can be viewed essentially as a deterministic overdamped system in a viscous medium. In sum, our computational model elucidates how various mechanical responses (thermal forces and the bending and stretching of actin filaments and ACPs) govern viscoelastic properties of the network under different conditions.

## Results/Discussion

### Model overview

**Figure 1:**
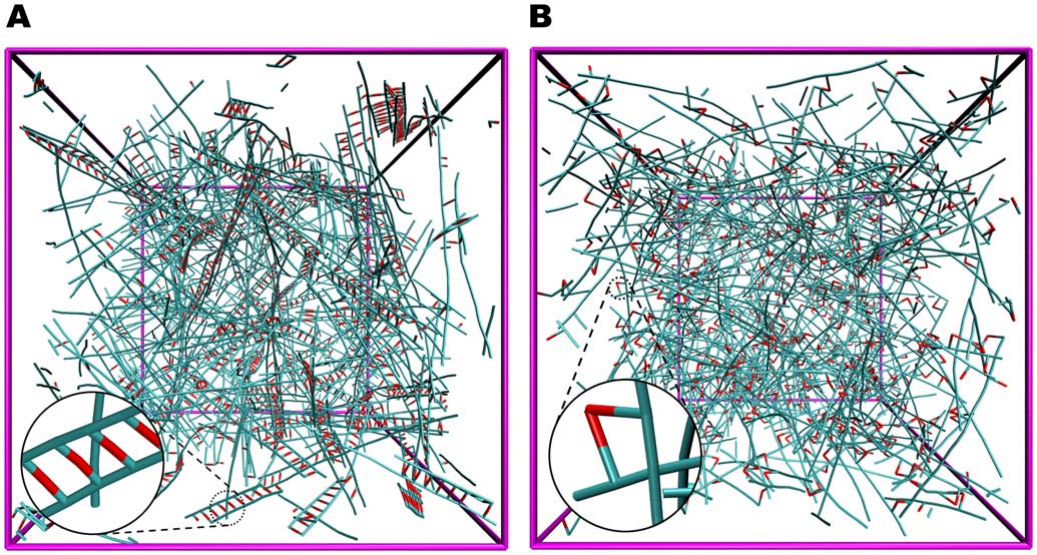
Two representative networks used in this study. (A) A network bundled via ACP^B^ and (B) crosslinked via ACP^C^, both of which consist of actin filaments of various lengths (cyan) and ACPs (red). For visualization, VMD was used [53]. Two arms of each ACP drawn with partial red and cyan are connected to filaments, forming crosslinks. In (A), due to the small computational domain, ladder-like structures are predominant rather than long, thick bundles. The linear dimension of the simulation box is 2.8 µm. These two networks form the basis for all simulations; networks with low *R* were obtained from these by eliminating a portion of active ACPs. The inset of each plot shows the detailed geometry of bundled or crosslinked structures consisting of actin filaments and ACPs.

To create an actin network, we began with a uniform distribution of actin monomers with ACPs dispersed randomly, and allowed the network to polymerize until 99% of the monomers were incorporated into filaments [35]. Motion of the monomers, filaments, and ACPs followed Brownian dynamics, and bonding occurred according to a first order irreversible process. After polymerization, we applied a coarse-graining procedure explained in Methods to cover a larger system size. In the resulting networks, the filament length distribution and the network morphology [35] bear a closer resemblance to reconstituted actin gels (Figure 1) than those generated by the random placement of equal-length filaments [24]–[26],[29],[32]. F-actins and ACPs in our model are characterized by introducing bending and extensional stiffnesses (Table 1). Two types of ACPs were used, depending on whether they form bundles (ACP^B^) or orthogonal networks (ACP^C^) during the polymerization process with equilibrium crosslinking angles of 0 and π/2, respectively, between the two crosslinked actin filaments.

**Table 1:** List of adjusted parameters.

Variable	Symbol	Value
Diameter of cylindrical actin segments	*σ*_C,A_	7.0×10^−9^ [m] (0.1)
Length of cylindrical actin segments	*L*_C,A_	7.0×10^−8^ [m] (1.0)
Time step	Δ*t*	6.17×10^−9^ [s] (2.0×10^−5^ )
Strength of repulsive force	*κ*_r_	1.69×10^−3^ [N/m] (2,000)
Extensional stiffness of actin filament	*κ*_s,A_	1.69×10^−2^ [N/m] (20,000)
Bending stiffness of actin filament	*κ*_b,A_	1.06×10^−18^ [N m] (255.0)
Concentration of actin	*C*_A_	1.21×10^−5^ [M]
Ratio of *C*_ACP_ to *C*_A_	*R*	0, 0.01, and 0.021 (ACP^C^)
		0, 0.01, 0.02, and 0.04 (ACP^B^)
*Extensional stiffness of ACP	*κ*_s,ACP_	4.23×10^−3^ [N/m] (5,000)
*Bending stiffness 1 of ACP	*κ*_b,ACP,1_	1.04×10^−18^ [N m] (250.0)
*Bending stiffness 2 of ACP	*κ*_b,ACP,2_	4.14×10^−18^ [N m] (1,000)

Numbers in parentheses are corresponding dimensionless values as defined in the text. The value and notation of other parameters are the same as in [35].

*: The same values are used for both ACP^B^ and ACP^C^.

Viscoelasticity of the network was probed in two ways: 1) In *segment-tracking rheology*, we analyzed the random thermal motion of many individual actin segments in the network [36]. 2) In *bulk rheology*, we applied an oscillatory shear strain on the top surface of the system while holding the bottom surface fixed. The measured viscoelastic moduli may depend on detailed geometry and the extent of percolation, especially if the network is small. Therefore, the use of a geometrically identical network for all simulations enables us to systematically control and isolate the effects of a given parameter. We thus used the two representative networks shown in Figure 1 for our measurements. The filament length (*L*_f_) was 1.5 µm±0.65 µm (average±standard deviation), and the actin concentration (*C*_A_) was 12.1 µM. We randomly removed ACPs from the networks to change *R*, the ratio of ACP concentration to *C*_A_ (parameters are defined in Table 1). Actin filaments longer than the width of the simulation domain (2.8 µm) were severed to reduce finite size effects.

### Comparison to experiments

**Figure 2:**
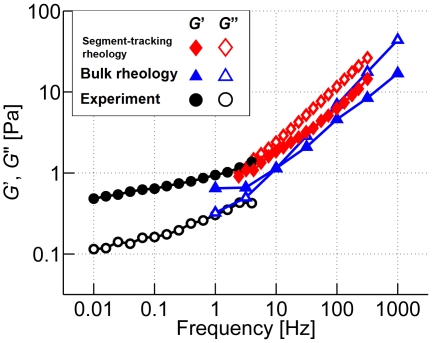
Comparison between computational and experimental results at <*L*_f_> = 1.5 µm, *R* = 0.01, and *C*_A_ = 12.1 µM. Solid symbols: *G*′, open symbols: *G*″. For simulation, bulk rheology (blue triangles) and segment-tracking rheology (red diamonds) were employed, while the experiment was conducted only using a bulk rheometer (black circles). Overlap occurs in the frequency range of 1–10 Hz.

While recognizing the limitations of any direct quantitative comparisons between our model predictions and experiments, we conducted one set of experimental measurements under conditions similar to those of the simulation (mean filament length <*L*_f_> = 1.5 µm_._, *R* = 0.01, and *C*_A_ = 12.1 µM) and compared viscoelastic moduli. In order to match <*L*_f_>, gelsolin was added to the sample; the length distribution was determined by fluorescence imaging. One difficulty in matching simulation and experimental conditions originates from the fact that *R* in the experiment corresponds to the total amount of ACPs in the sample, including both those in active and inactive (partially bound or free) states, whereas in our simulation, it indicates the net amount of active ACPs that crosslink or bundle two filaments. In addition, due to computational constraints, the oscillation frequency tested in the simulation overlaps that of the bulk rheology experiment only in a narrow range. Despite these difficulties, as seen in Figure 2, the values of *G*′ and *G*″ computed using the model are in reasonable agreement with the experiment, both qualitatively and quantitatively, perhaps better than one might have expected given the range of uncertainty of some of the parameters.

### Effects of the concentration and type of ACPs on *G*′ and *G*″

**Figure 3:**
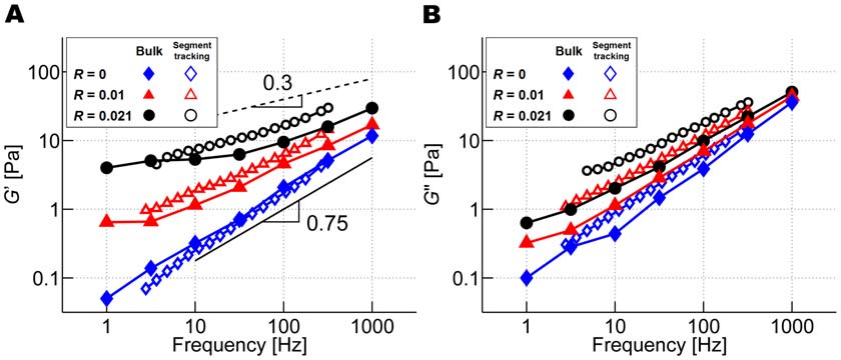
Viscoelastic moduli of networks crosslinked by ACP^C^. (A) *G*′ and (B) *G*″. Open symbols: segment-tracking rheology, solid symbols: bulk rheology. *R* = 0.021 (black circles), 0.01 (red triangles), and 0 (blue diamonds). With more ACP^C^, the magnitude of *G*′ increases, and its slope decreases. *G*″ is slightly larger for networks with higher *R*.

**Figure 4:**
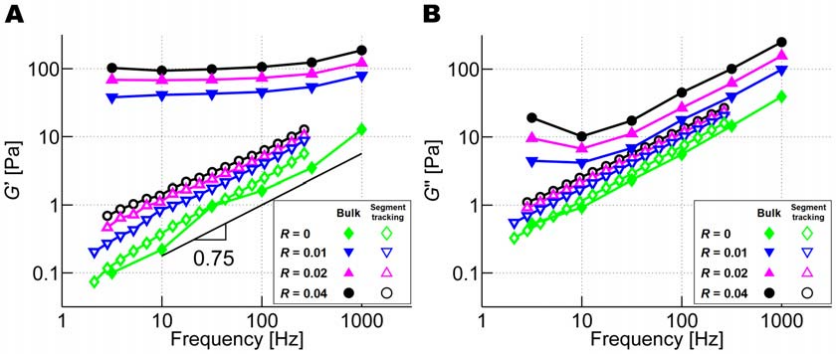
Viscoelastic moduli of networks bundled by ACP^B^. (A) *G*′ and (B) *G*″. Open symbols: segment-tracking rheology, solid symbols: bulk rheology. *R* = 0.04 (black circles), 0.02 (magenta triangles), 0.01 (blue inverted triangles), and 0 (green diamonds). Large discrepancies exist between results obtained by segment-tracking rheology and by bulk rheology with nonzero *R* due to heterogeneity of the bundled network.

We computed *G*′ and *G*″ of structures crosslinked via ACP^C^ (*R* = 0, 0.01, and 0.021) and those bundled via ACP^B^ (*R* = 0, 0.01, 0.02, and 0.04) using both bulk rheology and segment-tracking rheology. Networks without ACPs exhibit a slope of *G*′ close to 0.75, as indicated by the black solid line in Figures 3A and 4A. This value has been observed in various experiments [8], [37]–[39], and it is known to originate from transverse thermal undulations of actin filaments [30]. Interestingly, when repulsive forces between the filaments are eliminated, the slope of *G*′ estimated via segment-tracking rheology approaches unity (data not shown), implying that the volume exclusion effect of neighboring filaments creates a tube-shaped space that hampers free translation and rotation of the filament [31]. Although the filament confined in this way can perform reptation, it is operative on long time scales that are beyond those attainable in these simulations, so the mean square displacement (MSD) observed here primarily reflects transverse thermal motions, resulting in the 0.75 slope. On the other hand, the plateau in *G*′ often exhibited by experiments [6],[12],[40] was not observed within the frequency range of these simulations. Note that the plateau modulus is induced by entanglement effects that become more pronounced at longer time scales. The combination in these simulations of relatively short filaments leading to longer entanglement time [12] and computational constraints precluding simulations for longer times limited our ability to observe a plateau.

Values of viscoelastic moduli attained using bulk rheology and segment-tracking rheology exhibit surprisingly good agreement (Figure 3) even though segment-tracking rheology (Equation 11) was originally developed for a test particle much larger than the meshwork of filaments [36]. ACP^C^ elevates the magnitude of *G*′ and reduces its slope (Figure 3A), implying that the frequency dependence of *G*′ is reduced as networks incorporate more ACP^C^. *G*′ follows a power law, *G*′∼*f_s_* ^0.3^ (dashed line), for *f*_s_<100 Hz at the highest crosslink density, *R* = 0.021, which is within the range of powers observed in cells, 0.15–0.3 [9]–[11]. However, the magnitude of *G*′ from these simulations was much lower than *in vivo* values. This is likely due to many factors, notably the absence of prestrain. *G*″ increases slightly as the amount of ACP^C^ is increased, but the slope remains similar (Figure 3B). In addition, a decrease in the phase delay, tan^−1^(*G*″/*G*′) (Equation 10), accompanies the increase in *R*, indicating that ACP^C^ elevates the elasticity of the network. At *f_s_*∼10^3^ Hz, the phase delay depends only weakly on *R*, but as frequency decreases, it decreases more quickly with higher *R*, implying a greater effect by crosslinking at lower frequencies.

Networks bundled by ACP^B^ exhibit a behavior distinctly different from that of ACP^C^ (Figure 4). Large differences in *G*′ and *G*″ were observed between segment-tracking rheology and bulk rheology. This originates from the heterogeneity of the bundled network attributable to the small computational domain, for which viscoelastic moduli measured by bulk rheology depend strongly on whether or not there are bundles that percolate between the top and bottom boundaries. We thus discuss results of only segment-tracking rheology for networks formed by ACP^B^.

ACP^B^ increases *G*′ but has little effect on its slope in contrast to ACP^C^ (Figure 4A), whereas the phase delay is only slightly influenced by *R*. ACP^C^ is able to form a well-percolated network even at relatively low *R*, so that the network gels more efficiently [14] and acts like a single-body elastic object in response to shear stress or strain. By contrast, ACP^B^ bundles filaments together, resulting in the relatively low level of percolation for the same value of *R* as reflected by its low connectivity [35]. Although the diffusivity of bundled filaments is lower, the lack of connectivity leads to the absence of elastic behavior at long time scales. In order for ACP^B^ to increase network elasticity to a similar extent by ACP^C^, its concentration may have to be much higher.

### Effects of prestrain on *G*′ and *G*″

**Figure 5:**
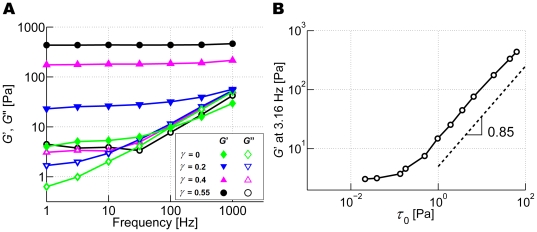
Behaviors of prestrained networks. (A) *G*′ (solid symbols) and *G″* (open symbols) of networks with *R* = 0.021 at various prestrain: *γ* = 0.55 (black circles), 0.4 (magenta triangles), 0.2 (blue inverted triangles), and 0 (green diamonds). At high prestrain, *G*′ becomes nearly independent of frequency. *G*″ with high prestrain slightly increases at low frequency. (B) *G*′ at *f*_s_ = 3.16 Hz versus prestress, *τ*_0_. *G*′ begins to increase at about 0.1 Pa and follows a power law, *G*′∼*τ*_0_^0.85^.

Only bulk rheology is employed here since the application of prestrain leads to a highly nonuniform distribution of the load, with a small fraction of highly tensed filaments and a larger number of filaments under little or no stress. Due to such heterogeneity, segment tracking rheology underestimates *G*′ and *G*″ as it randomly traces *N*_MSD_ segments from the entire network. The effect of prestrain on *G*′ is analogous to that of ACP^C^. As seen in Figure 5A, *G*′ increases and produces a weaker dependence on frequency at higher prestrain (*γ*); at *γ* = 0.55, *G*′ is virtually independent of frequency and is nearly 100-fold larger than that at *γ* = 0 for *f*_s_<10 Hz. This means that large prestrain transforms the network into a highly elastic one that exhibits a phase delay close to 0 at all frequencies and results in *G*′ comparable to *in vivo* values [9]–[11], wherein it has been postulated that prestress or prestrain plays a significant role [14]. *G*″ also exhibits interesting behavior; at high prestrain, it increases slightly at low frequency, similar to *in vitro* observations using heavy meromyosin (HMM) [18],[41], and a similar increase in *G*″ was also observed *in vivo* [19],[38]. This suggests that at low frequencies, viscous effects play an important role. Tharmann and coworkers argued that this trend of *G*″ may be due to the unbinding of HMM. However, since unbinding was not permitted in these simulations, the increase in the low-frequency *G*″ with prestrain must originate from a different mechanism. Note that such a tendency may have been more evident if the simulations were capable of reaching even lower frequencies.

Also, the relation between *G*′ at *f*_s_ = 3.16 Hz and prestress (*τ*_0_) was investigated. It remains relatively constant until a threshold prestress (*τ*_0_∼0.1 Pa, Figure 5B) beyond which it increases following a power law, *G*′∼*τ*_0_^0.85^. The exponent of 0.85 is close to the value of ∼1 found in *in vitro* experiments under similar conditions [14].

### Effects of extensional stiffness of actin filaments, *κ*_s,A_

**Figure 6:**
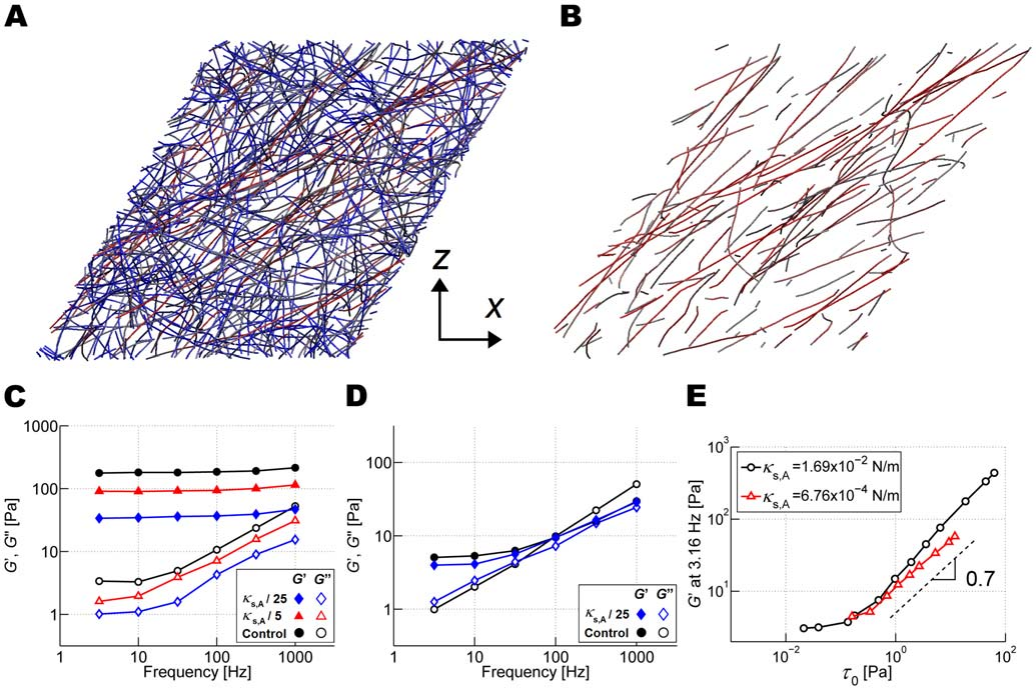
Importance and effects of extensional stiffness of actin filaments in prestrained networks. (A) Color-map of the prestrained network with *γ* = 0.55 (red: highly stretched bonds, gray: intermediately stretched bonds, blue: least stretched bonds.). (B) Similar plot showing only actin filaments with bonds stretched by more than 0.5%. (C,D) Influence of the extensional stiffness of actin filaments, *κ*_s,A_, on *G*′ and *G*″ for a network crosslinked by ACP^C^ (*R* = 0.021) at (C) *γ* = 0.55 and (D) *γ* = 0. Solid symbols: *G*′, and open symbols: *G*″ with *κ*_s,A_ = 1.69×10^−2^ (black circles), 3.38×10^−3^ (red triangles), and 6.76×10^−4^ N/m (blue diamonds) (E) *G*′ at *f*_s_ = 3.16 Hz as a function of prestress, *τ*_0_, for *κ*_s,A_ = 6.764×10^−4^ (red triangles) and 0.01691 N/m (black circles). *G*′ of both cases remains nearly constant at low prestress, but starts to increase above ∼0.1 Pa. The behavior is similar for the two values of *κ*_s,A_ except that lower *κ*_s,A_ leads to a slight reduction in both the level and slope (∼0.7, dashed line) of *G*′ above the threshold stress level.

To illustrate how prestrain transforms a network into a more elastic one, we display the network using a color scale depending on bond length averaged for duration of 0.1 ms. Only a small number of actin filaments aligned in the *x*-*z* direction are highly stretched (Figure 6A,B). As mentioned above, this heterogeneity precludes using segment-tracking rheology which measures thermal motions of randomly selected segments, many of which are not a part of the highly stretched filaments in prestrained networks. The mean filament length of the entire network increased by only 0.5% with *γ* = 0.55. However, due to the large value of *κ*_s,A_ (Table 1), this results in large spring forces that contribute significantly to the high magnitude of *G*′.

We also performed simulations with different extensional stiffness (*κ*_s,A_ = 0.0338 and 0.0068 N/m) for networks with *γ* = 0.55 (Figure 6C). *G*′ and *G*″ decreases with lower *κ*_s,A_, but its phase delay is virtually unchanged (data not shown). When *γ* = 0, however, variation in *κ*_s,A_ has little or no effect since most actin filaments are not highly stretched (Figure 6D). Therefore, *κ*_s,A_ affects viscoelasticity only under high prestrain.

We also studied the influence of *κ*_s,A_ on *G*′ (*f*_s_ = 3.16 Hz) at different levels of prestress (Figure 6E). Interestingly, when *κ*_s,A_ is reduced to 0.0068 N/m, the previous slope of 0.85 decreases to about 0.7, suggesting that the slope of the curve in the nonlinear regime increases with *κ*_s,A_. Note that we used a value of *κ*_s,A_ that is 1/40 of the experimentally measured value due to computational efficiency. Our result is thus consistent with a larger slope observed in experiments, ∼1 [14].

### Effects of bending stiffness and thermal fluctuation of actin filaments

**Figure 7:**
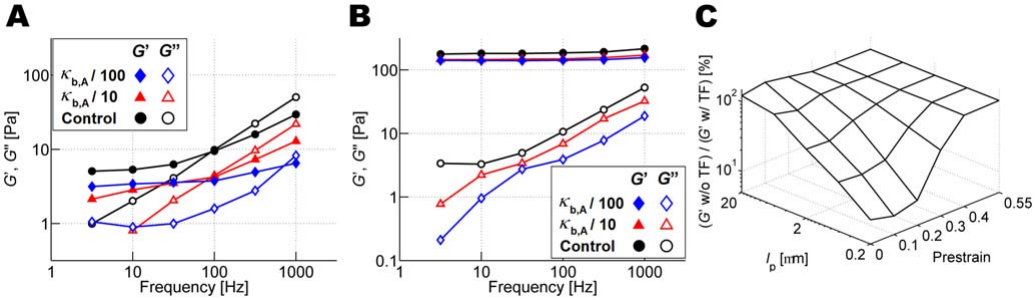
Effects of bending stiffness and thermal fluctuation on *G*′ and *G*″. (A,B) Effects of bending stiffness of actin filaments, *κ*_b,A_, on *G*′ (solid symbols) and *G*″ (open symbols) for a network crosslinked by ACP^C^ (*R* = 0.021) at (A) *γ* = 0 and (B) *γ* = 0.4. *κ*_b,A_ = 1.056×10^−18^ (black circles), 1.056×10^−19^ (red triangles), and 1.056×10^−20^ Nm (blue diamonds). The changes in *κ*_b,A_ have large effects on *G*′ and *G*″ in (A) but not in (B) (C) Effects of thermal fluctuation (TF) of actin filaments on *G*′ at *f*_s_ = 10 Hz as a function of *γ* and *l*_p_ (calculated at 300 K). At high *γ* (≥0.4) or large *l*_p_ (∼20 µm), TF plays no significant role in *G*′. On the contrary, at low *γ* and *l*_p_, *G*′ decreases without TF.

To address factors affecting viscoelasticity at low prestrain, we probed the influence of actin filament bending stiffness, *κ*_b,A_, by using three different values (*κ*_b,A_ = 1.056×10^−18^, 1.056×10^−19^, and 1.056×10^−20^ Nm) at *γ* = 0 (Figure 7A) and 0.4 (Figure 7B). At high prestrain, *γ* = 0.4, variations in *κ*_b,A_ have little effect on *G*′ and only minor effects on *G*″, suggesting that in the high prestrain regime, actin bending stiffness does not play a major role in viscoelastic properties (Figure 7B). By contrast, at *γ* = 0, *κ*_b,A_ influences *G*′ (Figure 7A), although perhaps not to the extent that one might expect for reductions in *κ*_b,A_ by factors of 10 and 100.

We also studied the effect of thermal fluctuation of actin filaments using separate simulations with or without the Langevin force term,**F** *_i_* ^B^ (Equation 1), at various *γ* and *l*_p_ (∼*κ*_b,A_/*k*_B_ *T*, with *T* = 300 K) (Figure 7C). Without **F** *_i_* ^B^, the temperature of the system drops to ∼10 K, as measured by applying the equipartition theorem, where the nonzero temperature is due to the externally imposed oscillation. Figure 7C shows the ratio of *G*′ without thermal fluctuation (TF) to that with TF at *f*_s_ = 10 Hz under each condition. At high prestrain (*γ*≥0.4), the elimination of TF does not affect *G*′ regardless of *l*_p_. Surprisingly, for filaments with bending stiffness comparable to that of actin (*l*_p_ = 10–20 µm), elimination of thermal effects had no significant effect on *G*′ at any value of *γ*. Only when *κ*_b,A_ was decreased (*l*_p_≤3 µm) did thermal fluctuations have a noticeable influence on *G*′. Under these conditions, *l*_p_ became comparable to the average distance between crosslinks, *l*_c_, (0.393 µm at *R* = 0.021), in which entropic effects would be expected to play a role [15], especially with a low prestrain. However, it is not clear whether this range of conditions can be attained *in vivo*. This finding that the relation between *l*_p_ and *l*_c_ determines the importance of thermal fluctuations is consistent with previous qualitative predictions [24]. Yet, our result is at odds with a previous view that entropic effects due to thermal undulations of individual filaments are responsible for the elasticity of scruin-crosslinked [15],[16] and HMM-crosslinked [18] networks even in the case when *l*_c_≪*l*_p_. In previous numerical studies [25],[26], thermal fluctuations were applied only before applying shear strain, not during the measurement of stress, and thus they cannot be used as a clear demonstration that thermal fluctuations are important. Further experimental, numerical, and theoretical investigations are necessary to clarify the role of thermal fluctuation and entropic elasticity. For instance, if network elasticity is mainly governed by enthalpy, and if thermal fluctuation plays little role, we expect that *G*′ will be minimally affected upon adding crowding agents to increase solvent viscosity and to provide steric barrier to conformational motion. However, crowding agents may affect organization of filaments, such as enhanced bundling [42]. Thus, such experiments will need to be carefully interpreted. Also, the vibrational motion of actin filaments depending on *l*_c_ measured in a computational study can be compared to theoretical predictions in [24].

### Effects of bending and extensional stiffnesses of ACPs

Previous studies showed that the detailed structure of ACPs strongly influences viscoelastic moduli of actin networks. In [20], each synthetically constructed crosslinking molecule of a different length produced distinctive macroscopic mechanical behaviors. In addition, Gardel et al. observed significantly different stress-strain relationships between a reconstituted actin network with intact filamin A (FLNa) and one with mutated hingeless FLNa [14].

**Figure 8:**
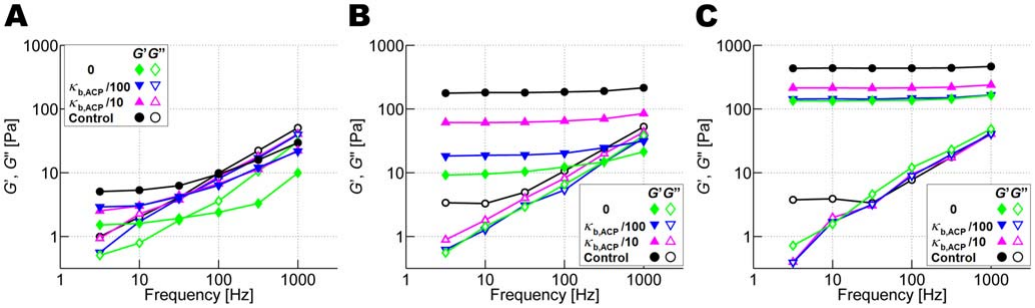
Effects of bending stiffnesses of ACP^C^, *κ*_b,ACP,1_ and *κ*_b,ACP,2_, on *G*′ and *G*″. The network is crosslinked by ACP^C^ with and without prestrain (*R* = 0.021). Solid symbols: *G*′, open symbols: *G*″. *κ*_b,ACP_ = control (black circles), 10-fold decrease (magenta triangles), 100-fold decrease (blue inverted triangles), and 0 (green diamonds) (A) *γ* = 0, (B) *γ* = 0.4, (C) *γ* = 0.55. The effects of changes in *κ*_b,ACP_ on *G*′ are the greatest in the network with *γ* = 0.4.

Since these previous results led us to anticipate substantial effects of ACPs in our actin networks, we investigated the influence of bending stiffness of ACP^C^, *κ*_b,ACP,1_ and *κ*_b,ACP,2_. As described in Methods, *κ*_b,ACP,1_ acts between two arms of the ACP, whereas *κ*_b,ACP,2_ limits bending between one arm of the ACP and the axis of actin filament to which it is attached, with an equilibrium value of π/2. To study their effects, we decreased both *κ*_b,ACP,1_ and *κ*_b,ACP,2_ 10 fold, 100 fold, and to zero. This had a significant effect on *G*′ and *G*″ at all prestrains (Figure 8), but especially so for the magnitude of *G*′ in the highly prestrained case with *γ* = 0.4. At small prestrain (*γ* = 0), deformation is mostly associated with the bending of actin filaments, and the ACP angles remain close to their equilibrium values. As prestrain increases (to 0.4), the actin filaments are progressively straightened, especially those that support the bulk of the load. Since this is accompanied by changes in the angle between crosslinked actin filaments, bending stiffness of ACPs becomes an important determinant of *G*′. As prestrain further increases (to 0.55), ACPs are maximally bent, and stretching of actin filaments is the dominant mechanism for resisting deformation since changing the extensional modulus has the greatest influence on *G*′. It should be noted, however, that though the geometry of ACP^C^ mimics that of FLNa, the large values of *κ*_b,ACP_ are closer to that of stiff scruin. Studies involving mutant ACP^C^ (e.g. FLNa) that differs in bending stiffness would further clarify our observation.

A change in extensional stiffness of ACP^C^, *κ*_s,ACP_, results in little change in *G*′ and *G*″ (data not shown) at low *γ*, but plays a significant role at high *γ*. This tendency is not surprising as unbending of the V-shaped ACP^C^ should precede stretch of ACP^C^ arms responding to the load.

### Significance of each parameter at various prestrains

**Figure 9:**
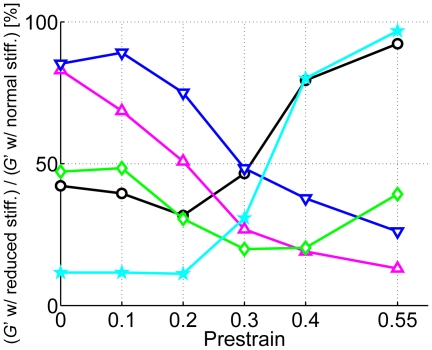
Relative decrease in *G*′ at *f*_s_ = 10 Hz due to 25-fold decrease of various stiffnesses at different prestrains. Magenta triangles: *κ*_s,A_/25, blue inverted triangles: *κ*_s,ACP_/25, green diamonds: *κ*_b,ACP_/25, black circles: *κ*_b,A_/25 with thermal fluctuation, and cyan stars: *κ*_b,A_/25 without thermal fluctuation. *R* = 0.021 was used. The influence of *κ*_s,A_ and *κ*_s, ACP_ increases at higher *γ*, and *κ*_b,ACP_ is significant at all prestrains. The effect of *κ*_b,A_ on *G*′ increases as *γ* decreases, and by comparing stars and circles, it can be inferred that thermal fluctuation plays an important role at very low *γ* when *l*_p_ is comparable to or less than *l*_c_.

Based on the effects of bending and extensional stiffnesses of actin filaments and ACPs as well as thermal fluctuations discussed above, we can estimate the relative importance of each factor over a wide range of prestrain and identify regimes where different phenomena dominate the viscoelastic behavior. For this purpose, each stiffness was decreased by 25-fold from the standard case, and the ratio of *G*′ with the decreased stiffness to that with the normal stiffness was computed at *f*_s_ = 10 Hz (Figure 9). A fall in extensional stiffness of both actin filaments (*κ*_s,A_) and ACPs (*κ*_s,ACP_) decreases *G*′ more as *γ* increases, implying that the stretch of filaments and ACPs plays an important role in strain-stiffening behavior at high *γ*, at least at the exaggerated levels of extensional compliance used in the simulations. Bending stiffness of ACPs, *κ*_b,ACP_, is significant at all tested *γ*, but has the largest effect on *G*′ for *γ*∼0.3–0.4. The influence of bending stiffness of actin filaments, *κ*_b,A_, on *G*′ is interesting, passing through a minimum at *γ* = 0.2. We measured *G*′ under the same conditions but without thermal fluctuation, and the minimum at *γ* = 0.2 disappeared; the ratios at *γ* = 0–0.2 are similar to each other. Therefore, thermal fluctuation of actin filaments contributes to an increase in *G*′ at low *γ* when *l*_p_ is comparable to *l*_c_.

Based on these observations, we propose three distinct regimes: i) A low *γ* regime where *κ*_b,A_ is dominant, with thermal fluctuations playing a substantial role for *l*_p_≤*l*_c_. ii) An intermediate *γ* regime in which the effect of *κ*_b,ACP_ becomes dominant. iii) A high *γ* regime where *κ*_s,A_ and *κ*_s,ACP_ are the predominant factors. Transition from one regime to another, however, is not sharply defined. Others [25],[26] have also argued that bending stiffness of filaments would dominate in the low *γ* regime, and extensional stiffness of filaments in the high *γ* regime, but they did not consider stiffness of ACPs as a parameter. Also, since these previous simulations lacked thermal fluctuations, their relative effects could not be assessed.

### The supportive framework governing viscoelastic moduli

**Figure 10:**
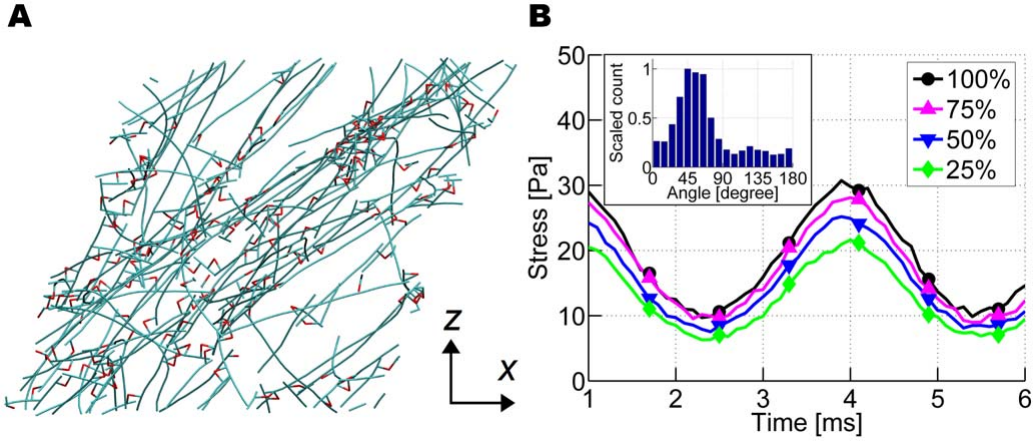
The supportive framework bearing most of stress. (A) Network composed of filamentous actin connected via ACPs that support the highest 25% of ACP bending forces. In contrast to Figure 6B, there are filaments that are almost perpendicular to the diagonal direction on the x-z plane, which are not highly stretched yet transmit load by bending of ACPs connected to them. (B) Stress exerted by prestrained networks (*γ* = 0.4) consisting of a fraction of actin filaments and ACPs. The extent of ACP bending forces is employed as a criterion to retain elements. Each symbol corresponds to a different percentage ratio of the number of ACPs remaining in a rebuilt network to that in the original network: 100% (black circles), 75% (magenta triangles), 50% (blue inverted triangles), and 25% (green diamonds). The fraction of remaining actin segments is, respectively: 100%, 79%, 52%, and 28%. (*inset*) Orientation angles of actin segments projected onto the x-z plane for the network in Figure 10A. Segments oriented in the z direction have a value of 0°. Most actin segments in the reduced structure are oriented in the (+x)-(+z) direction (45°), but segments with other orientations are also important, presumably because they transmit stress through bending of the ACPs attached to them.

Figure 6B shows that only a subset of the entire network supports the dominant portion of the stress in prestrained networks. While there could be many different criteria for identifying such a ‘supportive framework,’ we considered two, based either on the stretch of actin filaments or on the bending of ACPs. In the first case, we deleted filaments that were not highly stretched. The remaining network, however, when oscillated at the same strain amplitude, produced very low stress levels, suggesting that a significant fraction of the stress-supporting elements had been removed by this process. In the second case, we use bending force on ACPs as a selection criterion since the change in *κ*_b,ACP_ had a strong effect on viscoelastic moduli (Figure 8). First, the sum of magnitudes of all bending forces applied on each ACP in a prestrained state was calculated during 0.1 ms, the portion of ACPs with the highest bending forces were selected, and the remaining ACPs were deleted. All actin filaments not connecting between a pair of these highly strained and bent ACPs were removed. We compared stresses exerted on networks in which 25%, 50%, and 75% of ACPs remain under the same imposed prestrain. The network containing only 25% of initial ACPs (Figure 10A) consists of filaments oriented mostly in the diagonal direction on the x-z plane (*cf*., Figure 6B). Moreover, as the orientational distribution shows (Figure 10B, inset), there are (less stretched) filaments oriented in other directions that also help to transmit the applied load, by bending of connecting ACPs, which are not selected when a criterion based on stretch of actin filaments is used. The mean stress and oscillation amplitude of the reduced network remain nearly at original levels (Figure 10B). For example, the network containing only ∼28% of the original actin filaments and ∼25% of ACPs produced ∼70% of the original mean and amplitude of oscillating stress at the same levels of strain. Thus, bending force on ACPs is a major determinant of the elasticity of prestrained networks. We also confirmed that at high prestrain, ACPs experiencing large bending forces tend to displace in a manner consistent with the deformations being affine, as measured by the *S* parameter used in [43] (data not shown). This means that parts of the supportive framework that bear greater levels of force experience more affine deformation.

It is well known that most cells are in a prestressed state largely due to the actomyosin contractile apparatus [9],[44],[45]. By analogy, we hypothesize that the large *G*′ (∼10^3^ Pa) measured in cells is mostly due to the supportive framework composed of the contractile apparatus while the rest of cytoskeletal structures play comparatively little role in the macroscopic viscoelasticity of cells.

### Limitations resulting from permanent crosslinks

Most ACPs have finite binding lifetimes leading to dissociation and reformation of crosslinks, which is known to play an important role in the viscoelastic moduli and rheology of actin networks [22],[46],[47]. However, ACPs in our present simulation do not undergo dynamic rearrangement. This artifact renders the level of stress responding to prestrain unreasonably high. While stress catastrophically drops beyond 1∼30 Pa in experiments [14],[16],[20],[48], it can increase over 100 Pa in our simulation. The absence of crosslink dynamics causes *G*′ in Fig. 5A to be much higher and to exhibit a lower power compared to experiments. Nevertheless, our present results provide valuable insights as well as benchmarks whereby stress relaxation or dynamic reorganization of networks can be compared as an extension of this work.

### Conclusion

Using Brownian dynamics simulations, we systematically investigated viscoelastic moduli of actin-like networks. First, viscoelastic moduli measured in our model compared favorably with those of experiments conducted using a bulk rheometer under similar conditions. Then, effects of the kind and concentration of ACPs on *G*′ and *G*″ were investigated. With no ACP, *G*′ exhibited a power law slope of 0.75 against frequency, reflecting the transverse thermal motion of actin filamets. ACP^C^ tended to produce networks that were more elastic with a weaker dependence of *G*′ on frequency and smaller phase delay than ACP^B^. The influence of prestrain on *G*′ and *G*″ was also studied. High prestrain makes the network more elastic mainly via the stretch of a small fraction of aligned actin filaments. In addition, the effects of extensional and bending stiffnesses of actin filaments and ACPs on viscoelasticity were tested individually. We found that for viscoelastic moduli, three regimes are evident, each governed by different mechanisms depending on the amount of prestrain. At low prestrain, bending stiffness of ACPs and actin filaments plays an important role in determining viscoelasticity. At intermediate prestrain, bending stiffness of ACPs affects viscoelastic moduli the most. At high prestrain, the extensional stiffness of actin filaments and ACPs becomes dominant. We also found that entropic effects due to thermal fluctuations of filaments are important only when the prestrain is low and the average distance between active ACPs is comparable to the filament persistence length. Last, we identified the supportive framework that largely accounts for the high elasticity of prestrained networks, as that associated with strained ACPs rather than stretched actin filaments. Even after 75% of the network components are deleted, stress remains at 70% of the original value.

Our computational model using the discrete network of crosslinked F-actins provides insights into the microscopic origin of the viscoelastic behavior of the actin cytoskeleton. Importantly, our findings regarding the limited role of thermal fluctuation, as well as the different contributions to viscoelastic responses by the bending or stretching of F-actin or ACPs, require additional experiments for further investigation. The present computational framework can be further developed to study phenomena associated with ACP unbinding, such as stress relaxation, network reorganization, and plastic deformation by shear.

## Methods

Our Brownian dynamics (BD) model extends our previous work in which we investigated the morphology of polymerized and crosslinked actin networks [35]. In this simulation, actin monomers, filaments, and ACPs experience thermal motion and interact with each other with defined potentials and binding probabilities. Individual displacements are governed by the Langevin equation:
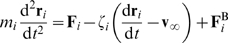
(1)where *m_i_* is the mass of *i*th element (actin or ACP), **r** *_i_* is the element's location, *ζ_i_* is the friction coefficient, *t* is time, and **v**_∞_ is the velocity of the surrounding medium. **F** *_i_* is a net deterministic force, and 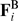 is a stochastic force satisfying the fluctuation-dissipation theorem. Considering that inertia of all elements is negligible on the length and time scales of interest, the Langevin equation is simplified by setting the acceleration term to zero and cast in nondimensional form using *k*_B_ *T*, *ζ*_C,A_, and *L*_C,A_ as primary variables, where *ζ*_C,A_ and *L*_C,A_ are the friction coefficient and length of a segment of F-actin (as explained later). Positions of all elements are updated using the Euler integration scheme:
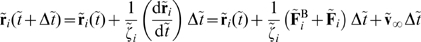
(2)where dimensionless variables are indicated by the tilde “^∼^”. Two interaction potentials describe bond stretch and bending with stiffness 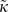, denoted by subscripts “s” and “b”, respectively [35]:
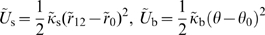
(3)where 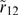 is the distance between two points constituting a chain, *θ* is the bending angle, and the subscript 0 denotes the equilibrium value.

### Coarse-graining scheme using cylindrical segments

In our previous model, we treated a segment of F-actin as a spherical particle representing two G-actins. To simulate larger length and time scales, we introduced coarse-graining in which a cylindrical segment represents several monomers. We kept thermal forces on ACPs and actin segments in a form similar to our previous model, but incorporated a cylindrical geometry for calculating repulsive forces.

**Figure 11:**
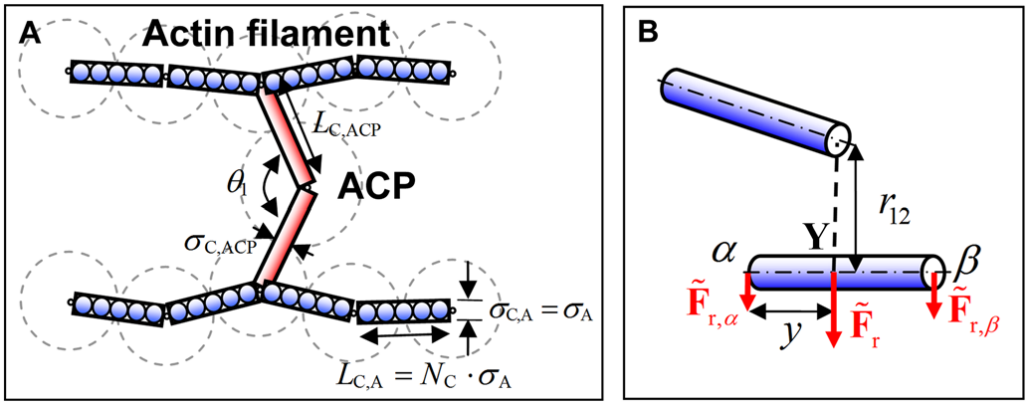
Schematic diagrams explaining the geometry of a network and the calculation of repulsive forces. (A) A coarse-graining scheme using cylindrical segments with *N*_C_ = 5. Dashed lines show monomers and ACPs used to generate the network using the polymerization model [35]. Once the network is formed, it is coarse-grained by replacing the original spheres by cylinders as shown. (B) A schematic diagram showing the distribution of the repulsive force acting on the point Y, 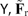 onto two end points, *α* and *β*. The proportion of each force is determined by *y*, the distance between point *α* and point Y.

As seen in Figure 11, the points of two adjacent elements on a filament correspond to the ends of one cylindrical segment representing *N*_C_ actin monomers of the previous model. Accordingly, the diameter of the cylindrical segment, *σ*_C,A_, is the same as the diameter of actin monomer of the previous model, *σ*_A_ = 7 nm, and its length, *L*_C,A_, is *N*_C_·*σ*_A_. By letting each monomer of the polymerized network evolve into a cylindrical segment, a larger network is created, albeit with a lower concentration of ∼1–100 µM. In addition, one point on a filament axis and the other point representing the center of an ACP determine the two end points of each arm of the ACP, indicating that ACPs have a structure with two cylindrical arms whose length and diameter are *L*_C,ACP_ and *σ*_C,ACP_ respectively. In this study, *N*_C_ was set to 10; this degree of coarse-graining is appropriate since the length of one rigid cylindrical segment, *L*_C,A_ = 70 nm, is still much shorter than the persistence length of an actin filament, ∼10 µm.

One consequence of this coarse-graining technique is that it increases the arm length of ACPs. That is, since the redundant volume of the original monomers is neglected, the arm length of ACPs is extended by (*L*_C,A_−*σ*_C,A_)/2. This produces a network for which the shortest distance between two bundled filaments is (*L*_C,A_−*σ*_C,A_)/2 (63 nm in this case) that exceeds the typical spacing formed by many of the bundling ACPs (e.g. fascin, fimbrin, and α-actinin). However, it is not expected that this will significantly alter the qualitative behavior of bundled networks since the change in the ACP arm length is much shorter than the length of F-actin. Previous experiments where different bundling ACPs led to distinct macroscopic behaviors [21],[22] are more likely due to the different stiffness and dissimilar binding affinities of the ACPs rather than their physical size.

Repulsive forces are computed according to the following harmonic potential depending on the minimum distance, 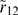, between two cylindrical segments, 1 and 2:
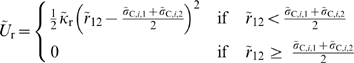
(4)where 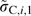 and 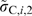 are diameters of the cylindrical segments (*i* = A or ACP), and 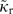 is the strength of repulsive effects. Forces calculated from the potential (Equation 4), 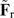, are distributed onto the two end points constituting the cylindrical segment, *α* and *β*, via the following equations:
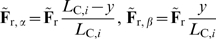
(5)where *y* represents the location on the segment at which the minimum distance, *r*_12_, is measured (Figure 11B; 0≤*y*≤*L*_C,*i*_).

### Polymerization and crosslinking

Monomer assembly and disassembly are important determinants of the morphology of actin networks. Given the rate constants obtained in recent *in vitro* experiments [49], it would take ∼100 s for an actin filament 1.5 µm in length to completely depolymerize, or ∼1–10 s to polymerize the same filament with *C*_A_ = 12.1 µM. Though *in vitro* depolymerization of F-actin is very slow, various ACPs can accelerate it *in vivo*. However, we assume here for simplicity that neither polymerization nor depolymerization of actin filaments occurs within the time scale of interest in this study, ∼1 s. Unlike our previous model [35], an ACP can bind to any point along an actin filament in any circumferential direction.

### Preparation of a network to estimate viscoelastic properties

The measurement of viscoelastic moduli can be influenced by the detailed geometry and the extent of percolation, especially if the network is small. Therefore, the use of a geometrically identical network for all simulations enables us to systematically control and isolate the effect of a given parameter. In other studies, actin networks have been generated by the random placement of equal-length filaments [24]–[26],[29],[32]. However, we generated more realistic networks using the previous model that incorporates both polymerization and crosslinking. A somewhat heterogeneous network bundled by ACP^B^ (Figure 1A) and a well-percolated, homogeneous network crosslinked by ACP^C^ (Figure 1B) were prepared. In Figure 1A, ladder-like structures consisting of two actin filaments and multiple ACP^B^ are evident, in contrast to thick bundles that are often observed in experiments. The relative absence of thicker bundles in these simulations is attributable to the small domain size compared to an average filament length. With *N*_C_ = 10, the filament length (*L*_f_) is 1.5 µm±0.65 µm (average±standard deviation), and the actin concentration (*C*_A_) is 12.1 µM. We randomly removed ACPs to change *R* (Table 1), while maintaining the overall network geometry. In addition, actin filaments longer than the width of the simulation domain (2.8 µm) were severed to minimize finite size effects.

### Adjusting parameter values for coarse-graining

Upon coarse-graining, several parameters need to be adjusted. For the cylindrical geometry of the segments, approximate forms for friction coefficients are [50]:
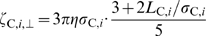
(6)

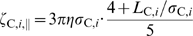
where *η* is the viscosity of the surrounding medium. However, since the mostly crosslinked cylindrical segments move predominantly in the transverse direction, and considering that 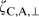 is only 1.64 times higher than 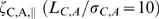, 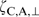 was used in all directions for simplicity. To test this assumption, we compared simulations with and without the anisotropic friction coefficient at zero prestrain. At *f*_s_ = 10 Hz, we obtained *G*′ = 5.07 Pa (isotropic) and 5.00 Pa (anisotropic), and *G*″ = 2.02 Pa (isotropic) and 2.39 Pa (anisotropic). Since motion along the filament axis is more suppressed with prestrain, the effect of anisotropic friction coefficient will be even less. We also assumed a constant friction coefficient regardless of the filament length to which the segment belongs. Hydrodynamic interactions between filaments are expected to play little role and were ignored since the volume fraction of actin is low (∼0.1%), and because the filaments have high aspect ratio (long thin rod) [51].

Since the geometry of ACPs changes after coarse-graining, the following equilibrium values for additional harmonic potentials were used for ACP^B^:
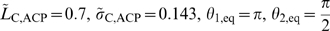
(7)

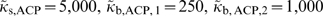
and for ACP^C^:
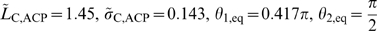
(8)

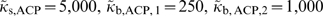
where 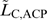 and 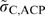 are the length and diameter of a cylindrical arm of ACP, 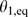 is the equilibrium angle between two arms of ACP, and 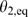 is the angle formed by an arm of ACP and the axis of the actin filament to which the ACP is bound. 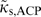, 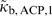, and 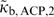 are stiffness constants related to 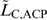, 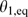, and 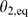, respectively (Figure 11A). The value of 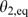
is the same as in the previous model, and 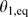 of ACP^C^ was adjusted to maintain an equilibrium minimal distance of 70 nm between two crosslinked filaments. 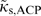 was set to be one fortieth that of an actin filament due to computational efficiency. 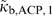 and 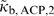 of ACP were estimated to be similar to the bending stiffness of an actin filament. Other parameters were also modulated according to the altered scale, as listed in Table 1. In our previous model by which the network was generated [35], a crosslink was allowed only if the torsional angle between two filaments was close to the equilibrium value (0 for ACP^B^ and π/2 for ACP^C^), and a finite stiffness was assigned to the torsional angle between crosslinked filaments. However, because the other two bending forces can preclude free torsional rotation, the torsional force is neglected here for simplicity.

### Bulk rheology – simulation

The concept of a strain-controlled bulk rheometer used in experiments was adopted in order to measure the viscoelastic moduli of the generated networks. First, all actin filaments were severed at the upper and lower boundaries, and the periodic boundary condition on those surfaces was deactivated. Cylindrical actin segments within 70 nm from the bottom surface were fixed, whereas those within the top 70 nm were forced to move following an imposed strain. For the application of prestrain, the top boundary was translated at a constant strain rate, 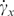, up to the desired strain. To measure differential viscoelastic moduli as in [14], a small sinusoidal strain (5%) was superposed on top of the finite prestrain. The sum of forces on the ends of filaments attached to the top boundary was calculated, and divided by the area of the top surface to determine stress. Only the force component parallel to the surface was considered. In addition, due to the small dimension of the system, the time scale for water diffusion through the computational domain is of order 10 µs. Since this is smaller than the smallest period of oscillatory strain, we assumed that the imposed shear strain immediately induces a linear velocity profile within the fluid. Consequently, after calculating the stress due to filament forces, we added a shear stress expressed as:
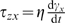
(9)where *τ_zx_* is the shear stress exerted in the x-direction on a plane perpendicular to the z-direction (pointing from the bottom to the top face), and *γ_x_* is the shear strain applied in the x-direction. The induced velocity of medium affects the movement of elements via the **v_∞_** term of Equation 2.

Finally, dividing the measured stress (*τ_zx_*) by the differential strain (*γ_x_*), viscoelastic moduli, *G*′ and *G*″, can be evaluated:
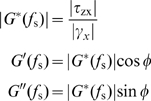
(10)where 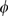 is phase delay between strain and stress, and 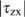 and 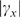 are the amplitude of the differential stress and differential strain, respectively. Note that 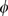 is zero for a perfectly elastic material and equals 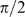 for a perfectly viscous one.

### Bulk rheology – experiment

We also measured the mechanical properties of *in vitro* F-actin networks with a rheometer (AR-G2, TA Instruments) using a 40 mm parallel plate geometry. Lyophilized actin monomer from rabbit skeletal muscle was purchased from (Denver, CO). To minimize artifacts caused by sample preparations [52], the actin was stored at high concentration (10 mg/ml) at −80°C and thawed rapidly at 37°C before each experiment. Recombinant filamin A was purified from Sf9 cell lysates, and recombinant human gelsolin was produced in *Escherichia coli*. Solutions of gelsolin, filamin, actin polymerization buffer, and actin were gently mixed. The solutions were then loaded within 10 s into a rheometer to form a crosslinked F-actin network. After 2 hr of polymerization at room temperature, frequency-dependent shear moduli, *G*′ and *G*″, were measured in the range of 0.1–10 Hz. To obtain mechanical properties in the linear elastic regime, strain was maintained below 2%.

### Segment-tracking rheology

Many experiments have used microbead rheology to probe viscoelastic moduli of actin gels based on the concept that the thermal motion of the bead is reflective of the gel's viscoelastic properties. Here, we used a variation of this approach, and tracked thermal fluctuations of individual actin segments. First, the domain was divided into a cubic lattice comprised of *N*_MSD_ cells (*N*_MSD_ = 512) of equal volume. Then, one cylindrical element was randomly selected per cell, and MSDs of the center of these elements were recorded over time. Using a well-known approximate method [36], *G*′ and *G*″ were calculated from the MSDs. Considering that this method was initially designed for a spherical bead, it was appropriately modified for cylindrical elements:
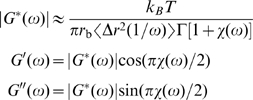
(11)where *r*_b_ is the effective radius of an actin segment, which satisfies 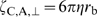. 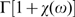 is the gamma function, where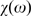 is the power law exponent describing the logarithmic slope of 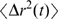 at 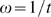, 
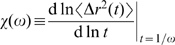
.

### Computational domain size and attainable time range

In spite of the coarse-graining using cylindrical segments, the length and time scales that our model can attain are still much smaller than those of usual experiments. For example, *L*_f_ in this computational model is a few microns at most, and the width of the 3-D domain is less than 5 µm. In such a small domain, it is difficult to investigate the effects of a wide range of *L*_f_ or *C*_A_ on viscoelastic moduli since *L*_f_ cannot be longer than the width of the domain to avoid artifacts associated with self-repulsion, and because an increase in *C*_A_ was achieved by a decrease in domain size with a constant number of molecules. If we increase the number of molecules, simulation time significantly increases. On the other hand, many *in vitro* studies have used quite long actin filaments (∼20 µm) with relatively large systems of size ∼O(1 mm). It takes about 16 days to reach 1 s in typical conditions using an Intel Xeon 2.66GHz CPU, but experimental results span up to 100–1000 s. Thus, an exhaustive comparison between computational and experimental results was not possible.
